# Identification of Genetic Variations in the NAD-Related Pathways for Patients with Major Depressive Disorder: A Case-Control Study in Taiwan

**DOI:** 10.3390/jcm11133622

**Published:** 2022-06-23

**Authors:** Daniel Tzu-Li Chen, Szu-Wei Cheng, Tiffany Chen, Jane Pei-Chen Chang, Bing-Fang Hwang, Hen-Hong Chang, Eric Y. Chuang, Che-Hong Chen, Kuan-Pin Su

**Affiliations:** 1 School of Chinese Medicine, College of Chinese Medicine, China Medical University, Taichung 404, Taiwan; rocket1025918@gmail.com; 2 Department of Psychiatry and Mind-Body Interface Laboratory (MBI-Lab), China Medical University Hospital, Taichung 404, Taiwan; swchengjames@gmail.com (S.-W.C.); tiffchen6@gmail.com (T.C.); peko80@gmail.com (J.P.-C.C.); 3 Graduate Institute of Biomedicine, College of Medicine, China Medical University, Taichung 404, Taiwan; 4 School of Medicine, College of Medicine, China Medical University, Taichung 404, Taiwan; 5 College of Arts and Sciences, Emory University, Atlanta, GA 30322, USA; 6 Department of Occupational Safety and Health, College of Public Health, China Medical University, Taichung 404, Taiwan; bfhwang@mail.cmu.edu.tw; 7 Graduate Institute of Integrated Medicine, College of Chinese Medicine, and Chinese Medicine Research Center, China Medical University, Taichung 404, Taiwan; tcmchh55@gmail.com (H.-H.C.); chehong@stanford.edu (C.-H.C.); 8 Department of Chinese Medicine, China Medical University Hospital, Taichung 404, Taiwan; 9 Master Program for Biomedical Engineering, China Medical University, Taichung 404, Taiwan; chuangey@ntu.edu.tw; 10 Graduate Institute of Biomedical Electronics and Bioinformatics, National Taiwan University, Taipei 100, Taiwan; 11 Department of Chemical and Systems Biology, School of Medicine, Stanford University, Stanford, CA 94305, USA; 12 An-Nan Hospital, China Medical University, Tainan 709, Taiwan

**Keywords:** kynurenine pathway (KP), major depressive disorder (MDD), nicotinamide adenine dinucleotide (NAD), single nuclear polymorphism (SNPs)

## Abstract

**Background and Objectives:** Nicotinamide adenine dinucleotide (NAD) is an important coenzyme in various physiological processes, including sirtuins (SIRTs) and kynurenine pathway (KP). Previous studies have shown that lower NAD levels can be indicative of increased risks of cancer and psychiatric disorders. However, there has been no prior study exploring the link between NAD homeostasis and psychiatric disorders from a genetic perspective. Therefore, we aimed to investigate the association of genetic polymorphism in the pathways of NAD biosynthesis with major depressive disorder (MDD). **Methods:** A total of 317 patients were included in the case group and were compared with sex-matched control group of 1268 participants (1:4 ratio) from Taiwan Biobank (TWB). All subjects in the control group were over 65 years old, which is well past the average age of onset of MDD. Genomic DNA extracted from patients’ blood buffy coat was analyzed using the Affymetrix TWB array. Full-model tests were conducted for the analysis of single nucleotide polymorphism (SNPs) in all candidate genes. We focused on genes within the NAD-related candidate pathways, including 15 in KP, 12 in nicotinate metabolism, 7 in SIRTs, and 19 in aldehyde dehydrogenases (ALDHs). A total of 508 SNPs were analyzed in this study. After significant SNPs were determined, 5000 genome-wide max(T) permutations were performed in Plink. Finally, we built a predictive model with logistic regression and assessed the interactions of SNPs with the haplotype association tests. **Results:** We found three SNPs that were significantly associated with MDD in our NAD-related candidate pathways, one within the KP (rs12622574 in ACMSD) and two within the nicotinate metabolism (rs28532698 in BST1 and rs3733593 in CD38). The observed association with MDD was significant in the dominant model of inheritance with marital status, education level, and body mass index (BMI) adjusted as covariates. Lastly, in haplotype analysis, the three associated SNPs consisted of one haploblock in ACMSD, four haploblocks in BST1, and two haploblocks in CD38. **Conclusions:** This study provides the first evidence that genetic variations involved in NAD homeostasis in the KP and nicotinate metabolism may be associated with the occurrence of MDD.

## 1. Introduction

**Figure 1:**
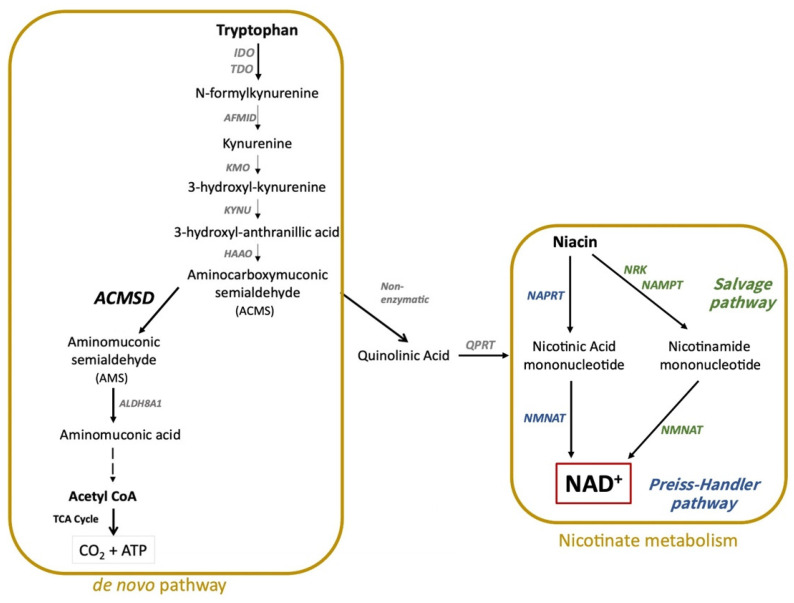
Brief summary of NAD^+^ biosynthesis pathways. NAD^+^ biosynthesis includes the de novo pathway (starts from dietary tryptophan) and nicotinate metabolism (Preiss-Handler pathway in blue and Salvage pathway in green, starts from niacin). Niacin includes nicotinamide, nicotinic acid, and nicotinamide riboside. Dietary tryptophan can be converted to either NAD^+^ or acetyl-CoA via the kynurenine metabolic pathway in mammals. ACMSD is the key enzyme that can moderate NAD^+^ homeostasis. Abbreviations: ACMSD, ACMS decarboxylase; AFMID, N-formylkynurenine formamidase; ALDH8A1, 2-aminomuconic semialdehyde dehydrogenase; CoA, coenzyme A; HAAO, 3-hydroxyanthranilate 3,4-dioxygenase; IDO, indoleamine 2,3-dioxygenase; KMO, kynurenine 3-monooxygenase; KYNU, kynureninase; NAD^+^, nicotinamide adenine dinucleotide; NAMPT, nicotinamide phosphoribosyltransferase; NAPRT, nicotinic acid phosphoribosyltransferase; NMNAT, nicotinamide mononucleotide adenylyltransferase; NRK, nicotinamide riboside kinase; QPRT, quinolinate phosphoribosyl transferase; TCA cycle, tricarboxylic acid cycle; TDO, tryptophan 2,3-dioxygenase.

Nicotinamide adenine dinucleotide (NAD), a vital coenzyme in redox reactions, regulates nutrients catabolism, energy metabolism, various metabolic pathways, and the behaviors in different species [1]. NAD alternates between the reduced (NADH) and oxidized (NAD^+^) states, and plays a key role in transferring electrons and protons in different metabolic pathways such as the tricarboxylic acid (TCA) cycle, glycolysis, fatty acid oxidation, gluconeogenesis, and biosynthesis of lipid and steroid. Processes of biosynthesis and degradation are therefore actively modulated by NAD [2]. In mammals, de novo biosynthesis of NAD is an integral pathway to dietary intake that uses tryptophan as the precursor. In addition, the Preiss-Handler pathway and the salvage pathway are also involved in the generation of NAD starting from niacin (nicotinic acid, nicotinamide, and nicotinamide riboside) as the precursor [3] (Figure 1). In contrast, the degradation of NAD involves several plasma membrane proteins such as CD38 molecule, bone marrow stromal cell antigen 1 (BST1 or CD157) dimer, and ecto-5′-nucleotidase (CD73): Zn^2+^ dimer. Besides the pathways of synthesis and degradation, phosphorylation of NAD also plays an essential role in human physiology. When ATP acts as a phosphoryl donor, NAD kinase catalyzes the phosphorylation of NAD and participates in the de novo biosynthesis of NADP [4]. Similar to NAD, NADP (the reduced form NADPH and the oxidized form NADP^+^) is also involved in numerous physiological responses associated with major depressive disorder (MDD) and postpartum depression (PPD) [5]. It is therefore important to also recognize how NADPH can affect the nervous systems through NAD in humans. Genome-wide association studies (GWAS) provide insights for the exploration of potential pathological genes in MDD. For instance, based on GWAS, genes such as Kynureninase (KYNU), Sirtuin (SIRT), glutamate ionotropic receptor AMPA type subunit 2 (GluR2), Neuronal Growth Regulator 1 (NEGR1), and High Mobility Group Box 1 (HMGB1) may relate to depressive symptoms [6,7,8,9,10,11]. Some of these aforementioned genes, including KYNU and SIRT, are also associated with NAD.

MDD is a severe psychiatric disorder, with more than 300 million patients worldwide. Even worse, it is the number one cause of disability [12]. The clinical expressions of MDD include two core features, depressed mood and loss of interest or pleasure in nearly all activities, and several accompanying symptoms, such as guilt, insomnia, anxiety, fatigue, weight loss and loss of appetite [13]. An epidemiology study revealed that the yearly and lifetime prevalences of MDD were 10.4% and 20.6% in the United States, respectively [14]. Another clinical trial reported that the average onset of the first MDD episode as 14.9 years old, with the duration ranging from two to 520 weeks [15]. Although serval antidepressants, such as selective serotonin reuptake inhibitors (SSRIs) and serotonin and norepinephrine reuptake inhibitors (SNRIs), are currently widely used, approximately 30% to 50% of patients do not respond fully and as many as 30% of patients are categorized as treatment-resistant [16,17]. Taken together, MDD is a serious public health concern that warrants more research to understand its pathology.

Previous studies have indicated that NAD deficiency may increase the risk of numerous disorders in human. For instance, it has been shown that metabolic function, longevity, and aging are modulated by a NAD-dependent histone deacetylase, sirtuin [18,19]. Sirtuin is also reported to be associated with multiple types of cancer [20] and disorder of circadian rhythm [21]. Importantly, it has been reported that 15–20% of the general population and 26% of the elderly suffer from NAD deficiency [22]. The prevalence is likely underestimated since NAD deficiency is often underdiagnosed clinically. Genetic variations that affect NAD levels in cell metabolism and epigenetic regulation may therefore play a role in human diseases. Previous studies, including clinical trials and animal models, suggested the link between NAD deficiency and several psychiatric disorders, such as chronic schizophrenia [23], anxiety disorder [24], and bipolar disorder type II [25]. NAD deficiency is also linked to MDD patients treated with antidepressants [26]. However, it seems that there have been limited genetic studies focused on the link between NAD homeostasis and psychiatric disorders, especially MDD. Although the association between MDD and sirtuin [27] and kynurenine pathway (KP) [28] has been widely reviewed previously, the association between MDD and NAD and its related pathways are still lacking.

α-amino-β-carboxy-muconate-epsilon-semialdehyde decarboxylase (ACMSD) is an important enzyme in the secondary pathway of KP, and is thus involved in tryptophan catabolism. Aminocarboxymuconic semialdehyde (ACMS), a toxic intermediate from the catabolism of tryptophan, is metabolized either non-enzymatically to quinolinic acid (QA) or is catalyzed enzymatically by ACMSD to acetyl coenzyme A (CoA). QA is converted to nicotinic acid mononucleotide by quinolinate phosphoribosyl transferase (QPRT) and converges with the Preiss-Handler pathway. Based on this metabolism, it was proposed that the activity of ACMSD may serve as a useful biomarker for the levels of NAD (Yoshino, 2019 [3]). Palzer et al. showed that overexpression of ACMSD resulted in deficiency of NAD in an acquired niacin dependency (ANDY) transgenic mice model [29]. In agreement with the findings from the ANDY mice, body weight and fat loss, and observation of low activity in open field, are reflective of depressive symptoms in patients with MDD. However, the similarities noted in animal models were not sufficiently strong to support MDD treatment clinical trials based on NAD supplement or boosters developed from NAD-related pathways. To establish a stronger link between NAD level and MDD, here we have used a genetic approach to investigate the associations between MDD and the NAD-related pathways, including tryptophan catabolism and nicotinate metabolism in humans.

## 2. Materials and Methods

In this case-control study, several candidate genes were selected based on the NAD-related pathways, including KP and nitroamide metabolism. We also investigated the genetic markers SIRTs and aldehyde dehydrogenases (ALDHs) which might be related to the development of MDD. SIRTs are NAD-dependent deacetylases and ALDHs are responsible for the removal of toxic intermediate aldehyde products. It has been shown that both genetic markers play essential roles in the NAD-related pathways and may be associated with the pathology of MDD [27,30]. Therefore, all SIRTs and ALDHs genes were also included in our search. Genotyping, searching strategy, permutation, and statistical analyses were applied to this study as described in our previous publication (Cheng et al., 2021 [10]).

### 2.1. Participants Selection and Cohort Building

Adult patients diagnosed with MDD based on the Diagnostic and Statistical Manual of Mental Disorders, Fifth Edition (DSM-V) [31] and with Hamilton Depression Rating Scale (HAM-D) score of >18 were enrolled in this study for genotyping. Patients with records of psychiatric disorders (such as bipolar disorders and schizophrenia), suicidal ideations, or substance use disorder were excluded from this study. A total of 327 patients who fit the inclusion criteria were enrolled in the case group of our study. As for the control group, we conducted sex-matched healthy controls to cases in a 4:1 ratio by using the data from Taiwan Biobank (TWB). In accordance with genotypes and single nucleotide polymorphisms (SNPs), we selected healthy participants over 65 years of age from TWB who were more likely to have passed the average age of onset (AOO) of MDD [32]. All subjects enrolled in this study were consented and provided with educational information on MDD following an approved IRB protocol for this study. Treatments such as antidepressants and other psychosocial therapies were also provided.

### 2.2. Genotyping

Genomic DNA from the patients was extracted from the buffy coat using QIAamp DNA Blood Mini Kit (Qiagen, Crawley, UK). Genotyping was performed using the Affymetrix TWB array, an axiom genome-wide array plate supplied by Thermo Fisher Scientific (MA, USA) at the National Center for Genome Medicine in Academia Sinica, Taipei, Taiwan. Each single array chip contains over 600,000 SNPs, including genotyped and imputated SNPs. The SNP source comes from the clinical and research human genome database of Thermo Fisher Scientific, including the results of international research projects such as the HapMap Project and the 1000 Genomes Project. The array is adaptable and can be adjusted for different human populations using ethnic-specific chips.

### 2.3. Data Quality Control

We utilized Plink v1.90b6.24 for genome scale and SNP quality control (QC) of the subjects. The QC of the subjects included gender confirmation and clinical data management, such as MDD diagnosis and related medical surveys. Subjects with ambiguous clinical data or mismatch between assigned sex and chromosomal sex were excluded. No further exclusion was performed after QC. As for the QC of SNPs, data retained in our study were: (1) genotyping rate ≥ 95%, (2) minor allele frequency (MAF) ≥ 0.01, and (3) Hardy-Weinberg equilibrium with a significance level of 0.001 in controls, as calculated by the exact test (Wigginton et al., 2005 [33]). The total successful genotyping rates were 99.7% in cases and 98.7% in controls. Accordingly, 317 out of 324 subjects in the MDD case group passed the QC. We then matched the data against 1268 (4 times of cases) healthy controls, all of which passed the QC test as described above.

### 2.4. Statistical Analysis

The software used for statistical analysis was Plink v1.90b6.24. Analysis was performed to investigate the correlation between MDD and the candidate genes. Case-control association analysis using allelic, dominant, and recessive models was applied to identify the significant SNPs among all candidate SNPs. A genome-wide max(T) permutations of 5000 strengthened the control over genome-wide familywise error rate through creating empirical *p*-values. In this process, we compared the best original result of each significant SNP against the others. The genome-wide permutations process powered by an accelerated expectation-maximization algorithm [34] or SNPs and haplotypes has been recognized as a valid predictor for the effects of multiple genes and linkage disequilibrium [35,36]. For instance, previous studies provided evidence suggesting the possible pathology of the kynurenine pathway [10] and the ionotropic glutamate receptor pathways [11] in MDD. Furthermore, to evaluate the combination effect of each SNP in the same gene, we carried out the haplotype association tests with Haploview 4.2. At last, we performed logistic regression for each of the significant SNP found to build our predictive models. On top of that, the significant characteristics of our subjects, including marital status, education level, and body mass index (BMI), were chosen as appropriate covariates in the logistic predictive models.

## 3. Results

### 3.1. Candidate Gene Selection

We utilized the Reactome pathway database (https://reactome.org/, accessed on 23 October 2021) to select our candidate genes in the pathways that may be involved with NAD biosynthesis, degradation, metabolism, and homeostasis. Following a search with the keyword “Tryptophan”, two pathways were identified: tryptophan catabolism (or KP, R-HSA-71240), and its secondary pathway nicotinate metabolism (R-HSA-196807). The results were suggested and supported by additional database search, including Kyoto Encyclopedia of Genes and Genomes (KEGG) and Gene Ontology (GO). Additionally, we utilized the Protein Variation Effect Analyzer (PROVEAN), a validated silico tool for the functional prediction of protein (Choi et al., 2012 [37]), to confirm our findings. In total, there were 15 candidate genes in KP and 12 candidate genes in nicotinate metabolism. In terms of the genetic markers, all 7 SIRTs genes and 19 ALDHs genes were included in our search. The representative variants that may displayed the functional or structural changes, such as tag SNPs, to all candidate genes were included. A total of 508 SNPs were analyzed in this study. There were 166, 126, 22, and 194 SNPs after QC in KP, nicotinate metabolism (Table 1), SIRTs, and ALDHs (Table 2), respectively. Detailed information, such as variation type, allele frequency of genes, and SNPs information are shown in Supplementary Tables S1–S4.

### 3.2. Full-Model Case-Control Association Test

The Full-model association test includes allelic, dominant, and recessive models. SNPs in the candidate pathways with an empirical *p*-value < 0.05 obtained by a permutation test were considered significant. We found three significant SNPs, all of which were based on the dominant models. They were ACMSD in tryptophan catabolism, and BST1 and CD38 in nicotinate metabolism. The significant SNPs are rs12622574 in ACMSD (empirical *p*-value = 0.0256), rs28532698 in BST1 (empirical *p*-value = 0.0124), and rs3733593 in CD38 (empirical *p*-value = 0.0017). The MAFs were 0.1562, 0.0883, and 0.2437 in the cases, and 0.2078, 0.0607, and 0.3074 in the controls, respectively. Interestingly, rs3733593 has higher MAF in the cases while rs22622574 and rs28532698 have higher MAF in the controls. This result may be associated with the regulation of gene expression provided by the variations, such as intron-mediated enhancement (IME) or intron-mediated inhibition (IMI). All significant SNPs are introns, suggesting that structures of the enzymes in variations remained unchanged. Similar results for protein function were also obtained using the PROVEAN. However, no significant finding was noted in SIRTs and ALDHs (Table 3).

### 3.3. Logistic Regression

The dominant model was chosen based on our results as well as the suggestions proposed by Liu et al. (H. M. Liu et al., 2021 [38]). Logistic regression analysis showed significant associations in all the significant SNPs: rs12622574 in ACMSD (*p*-value = 0.0123; coefficient t-statistic (STAT) = −2.504; odds ratio (OR) = 0.712), rs28532698 in BST1 (*p*-value = 0.0104; STAT = 2.562; OR = 2.562), and rs3733593 in CD38 (*p*-value = 0.0042; STAT = 2.562; OR = 0.696). This result indicates that these three SNPs may be associated with the occurrence of MDD in Taiwanese, and further molecular level investigations are warranted.

As for the covariate analyses, a significant difference was only found in BMI (mean in the cases = 22.67, mean in the controls = 24.18, *p*-value < 0.001) between our cases and controls. This result may be due to the common clinical symptoms, i.e., weight loss, in patients with MDD. However, BMI did not show a significant effect on all of the significant SNPs, indicating BMI was likely not a predictive factor for these SNPs in patients with MDD.

### 3.4. Haplotype Analysis

**Figure 2:**
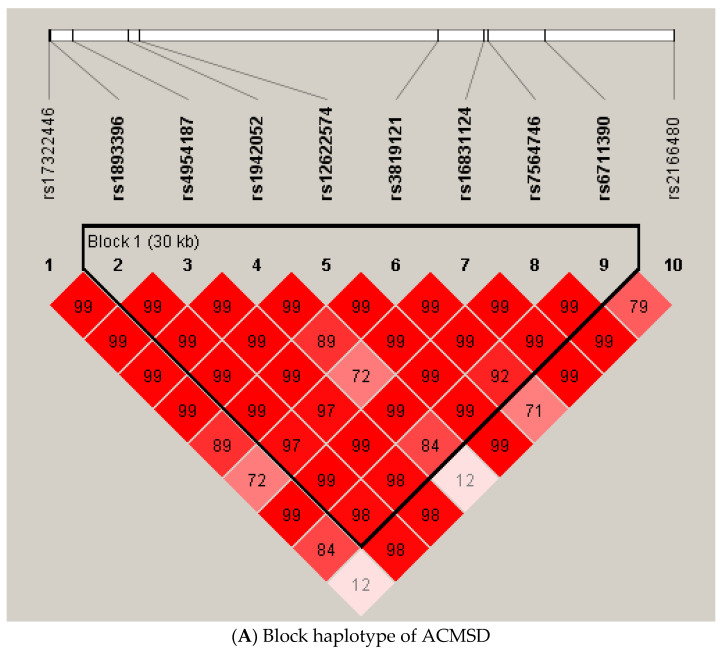

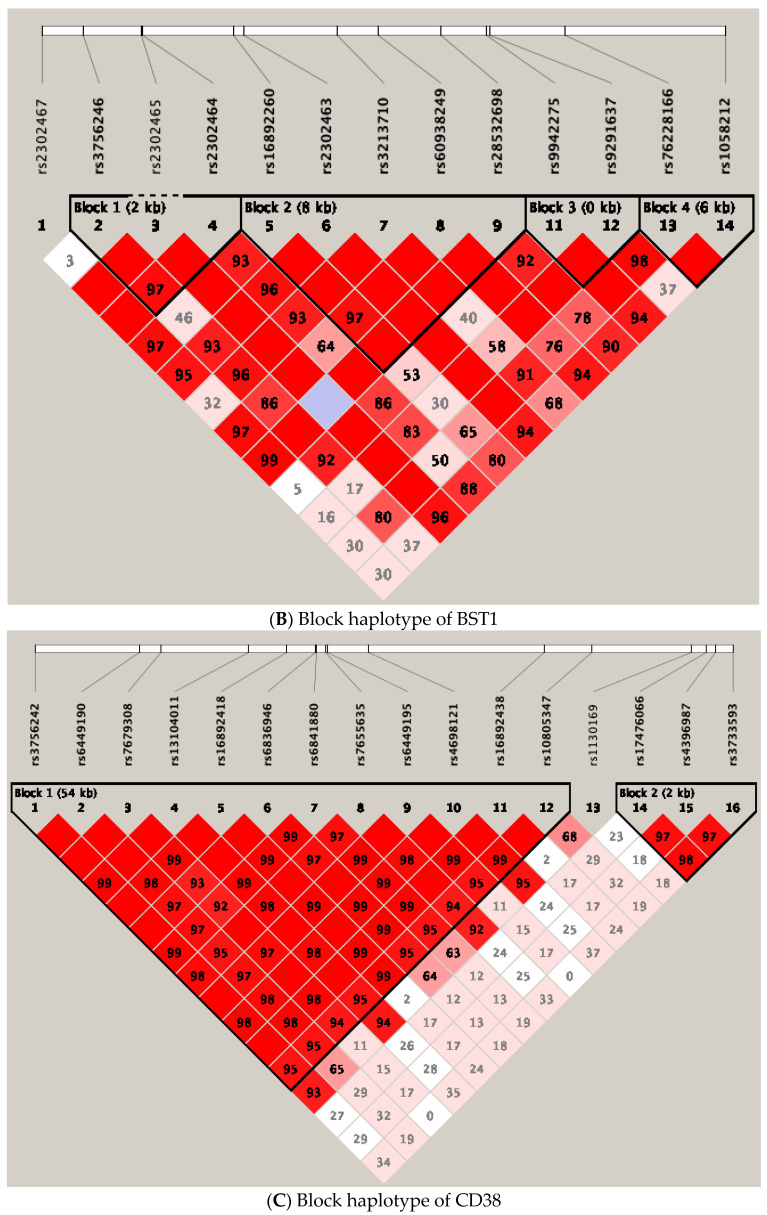
Haplotype Analyses of ACMSD, BST1, and CD38.

In order to investigate the combined effect for each of the three significant SNPs, haplotype association tests were conducted. According to the limitation of genotyping methods, some SNPs may be combined with other SNPs located near the original SNPs. Therefore, haplotype analysis is essential in genetic studies [39]. SNPs in the same haploblock (as defined by Gabriel et al. [40]) were determined by Haploview. We found one block in ACMSD, four blocks in BST1, and two blocks in CD38 with the haplotype association test. On top of that, one, two, and two significant blocks of ACMSD, BST1, and CD38 were identified in the haplotype analysis, respectively. They are TTTACATT in ACMSD (*p*-value = 0.0045), GCGCC (*p*-value = 0.0124) and GC in BST1 (*p*-value = 0.0050), and TCA (*p*-value = 0.0022) and TCG (*p*-value = 0.0134) in CD38 (Table 4 and Figure 2). However, there was only one block in each gene that was considered significant after permutation, which were TTTACATT in ACMSD (permutation *p*-value = 0.0256), GC in BST1 (permutation *p*-value = 0.0476), and TCA in CD38 (permutation *p*-value = 0.0230).

## 4. Discussion

To our knowledge, this is the first candidate gene study to examine the association between MDD and genetic variations in the NAD homeostasis-related pathways in the East Asian population. Our findings did not further support the results from previous GWAS, likely because of the differences in ethnicity between Asian and Caucasian populations, where the latter have been more widely conducted. Therefore, our observations may serve as a stepstone for future investigations that use large-scale GWAS targeted at the East Asian population. The strength of our study is the relatively larger size of the sex-matched control group with 65 years old and older. The lifetime prevalence investigation of mental disorders reported that the first onset of half of all cases started by the age of 14 and three fourths by the age of 24 [32], indicating the average age of onset (AOO) is in the childhood or adolescence among the majority of patients with MDD. In order to focus on the effect of genetic polymorphisms, the age threshold we performed can minimize the effect of other factors that may be associated with the onset of MDD.

Our first main finding is that a SNP in the ACMSD gene was found to be associated with MDD when compared to the control group. ACMSD is a key enzyme in tryptophan catabolism that regulates the biosynthesis of acetyl CoA and NAD. Our finding supports the link between MDD and the function of ACMSD and its correlation with NAD deficiency. Palzer et al. reported that overexpression of ACMSD may trigger NAD deficiency in an animal model [29]. The SNP, rs12622574, that we have identified is an intron variant. It is therefore not predicted to have any influence on the structure of the protein product. One possible reason is that it may be in linkage disequilibrium (LD) with other functional SNPs. Alternatively, the regulatory effect of introns, such as intron-mediated enhancement (IME), was widely reported in other studies, indicating that introns may modulate gene expression by affecting the efficiency or the stability of both transcription and translation [41,42]. Unfortunately, there has been no study focusing on the regulatory effects of rs12622574 on ACMSD expression so far. The allele frequency of rs12622574 in East Asian based on gnomAD human genome database (https://gnomad.broadinstitute.org, accessed on 25 February 2022) is 0.187, which is much higher than those in other populations. Future studies are therefore needed to investigate the regulatory effect of the intronic rs12622574 variant and the association between the ACMSD expression level or its enzyme activity and MDD, especially in the East Asian population.

Overexpression of ACMSD activity may lead to a buildup of 2-aminomuconate semialdehyde (AMS), a toxic metabolite of ACMS. AMS is first metabolized by either 2-aminomuconic semialdehyde dehydrogenase or aldehyde dehydrogenase 8A1 (ALDH8A1) isozyme to aminomuconic acid (AMA), then it enters one of the acetyl CoA biogenesis pathways (Figure 1). Due to the reactivity and toxicity of aldehydes, AMS is normally removed quickly by ALDHs. This implies that the variants of the ALDH8A1 or other ALDH isozymes may also play a role in maintaining the balance of NAD levels. A total of 1350 common ALDH genetic variants have been compiled recently based on the gnomAD human genome database [43]. Therefore, we investigated a selected panel of the common East Asian ALDH variants between our MDD cases and the healthy controls. No significant association with the ALDH variants was found after permutation. However, rs73393881, an intron variant of ALDH1L2, showed a trend of significance (empirical *p*-value = 0.05379, OR = 0.4803) between the cases and the controls after permutation, while all 9 SNPs, after QC, in ALDH8A1 SNPs did not. ALDH1L2 is a mitochondrial form of 10-formyltetrahydrofolate (10-CHO-THF) dehydrogenase [44]. In the presence of NADP^+^, it converts 10-CHO-THF, an aldehyde, to tetrahydrofolate (THF), the active form of folate. Accordingly, the bioactivity of ALDH1L2 may be essential to THF level. THF involves the biosynthesis of purines and pyrimidines, which are crucial during pregnancy [45]. Genetic evidence showed that folate deficiency may be a risk of PPD for women with a deficient MTHFR C677T TT genotype [46]. In addition, a cohort study reported that prolonged folate supplementation may decrease the risk of PPD [47].

CD38 and BST1 both play an essential role in the degradation of NAD, of which two significant SNPs (rs3733593 and rs28532698, respectively) were found to be associated with MDD. Both SNPs are also intronic, indicating that it may be the expression levels rather than the structure of proteins that leads to the association between these variants and the disease. CD38, a surface cyclic ADP ribose hydrolase, is expressed ubiquitously, especially in immune cells, while BST1 is expressed mainly in lymphoid tissue and gut [48]. Both CD38 and BST1 are glycohydrolases that cleave NAD to generate nicotinamide [49] so that NAD can be rapidly recycled via the salvage pathway. Previous animal studies showed that mice treated with CD38 inhibitors had 50% higher levels of NAD [50]. Moreover, CD38 knockout mice resulted in a more stable maintenance of the NAD level and were resistant to the negative effects of high-fat diets. Whereas mice overexpressing CD38 showed lower levels of NAD, defective mitochondria, decreased oxygen consumption, and increased lactate production [51]. Interestingly, CD38 inhibitors, such as apigenin, have been used as potential drugs for treating metabolic disorders [52]. In this study, the MAF of rs3733593 in the cases (0.2437) is significantly lower than that in the controls (0.3074). This result may support that the overexpression of CD38 is associated with MDD, and that the IME effect may have been presented. On the other hand, BST1 knockout mice showed anxiety and depression-like behaviors when compared to wild-type mice [53,54]. Interestingly, an antidepressant effect was observed when BST1 knockout mice were treated with selegiline, a monoamine oxidase-B (MAO-B) inhibitor [55]. However, there has been no clinical study targeted on BST1 and MDD so far. In our result, the MAF of rs28532698 in the cases (0.0883) is significantly higher than that in the controls (0.0607), indicating a possible IMI effect on BST1 of this SNP. The nature distribution in East Asian, based on GnomAD human genome database, of these two SNPs are 0.06859 (rs28532698) and 0.3082 (rs3733593), respectively. The population frequency of rs3733593 is relatively homogeneous in different ethnic groups, while rs2853298 has a much higher allele frequency in African/African American (0.3804) and Ashkenazi Jewish (0.1793). Therefore, our findings may not merely be important for MDD patients in East Asian, but in other populations as well. Up to now, there has been no study focusing on these two SNPs. It is possible that the SNP variants that we have discovered may be useful biomarkers for differential diagnoses and hold the potential for personalized drug choices for MDD in the future. On the other hand, despite the widely reported results stated previously, we did not find any SNP in the sirtuins genes associated with MDD. Further investigations on the association between SIRTs and MDD with a larger patient population should be conducted in Taiwan.

In clinical settings, NAD deficiency is often under-recognized because of the common symptoms it shares with MDD. Although there are some clinical justification and applications in regulating NAD level in several diseases [51,56], such as CD38 inhibitors for boosting NAD in children with spectrum disorders [57], the evidence for NAD and MDD is still limited. Therefore, genetic evidence is still needed, especially SNP variants to indicate a clear relationship between NAD and MDD. In this study, we discovered three new predictive SNPs in the NAD-related pathways with MDD, indicating a possible mechanistic link between NAD deficiency and MDD. These newly identified SNPs in ACMSD, BST1, and CD38 may serve as potential biomarkers for clinical differential diagnoses and as potential treatment targets for the modulation of NAD level in MDD patients carrying these SNPs. Our finding was in accordance with previous clinical studies which reported that antidepressants are associated with NAD deficiency, especially in patients with inadequate amino acid intake [58]. Thus, it is important to also consider how SSRI, SNRI, and tricyclic antidepressants may affect the NAD level and influence the immune system by regulating immune reactivity [59], altering cytokine responses [60], and inhibiting immune cells and the KP as a result of its anti-inflammatory effects [26,61]. However, we cannot clarify the combined effects from the genetic and pharmacology level; further studies are warranted.

Our study has several limitations. Firstly, except for the sex, age, marriage status, BMI, and education level, other sociodemographic factors (such as stress events from social environment or childhood, nutrients status, antidepressant interventions, and interpersonal relations problem) which may be associated with the onset of MDD were not included in our dataset. As a result, some potentially important variables may be missing. Secondly, the NAD status of participants was not measured in this study. NAD levels can be helpful in ascertaining the effects of SNPs in NAD biosynthesis, since epigenetic silencing of mutated genes may occur. The interaction between SNPs and NAD level should therefore be investigated further in future studies. Thirdly, the statistical power may be reduced due to LD within genes when using the Bonferroni correction. To compensate for this effect, we performed the permutation technique, a method widely accepted in genetic association studies, to avoid the high Bonferroni penalty [62]. The application of gene-set analysis to address the small effect sizes of individual markers in psychiatric disorders should also be considered in future studies. Moreover, the small sample size of our study is another limitation to strongly suggest the association between MDD and the new SNPs. Lastly, in order to eliminate false positives, we minimized the number of candidates. Our candidate pathways are relatively small, with suitable and relevant targets of only 15 genes in KP, 12 genes in nicotinate metabolism, 7 genes in SIRTs, and 19 genes in ALDHs in this genetic study. Since these SNPs have never been discovered in previous studies of MDD, they are novel targets that will need to be validated by future studies.

## 5. Conclusions

We found three significant variants (rs12622574 in ACMSD, rs3733593 in CD38, and rs28532698 in BST1) in the NAD-related pathways among patients with MDD. Our results provide potential genetic-level evidence for personalized medicine in patients with MDD, especially for the East Asian population. Moreover, the regulation effect of intron should be further investigated in future studies.

## Figures and Tables

**Table 1:** Candidate genes information in kynurenine pathway & nicotinate metabolism.

Pathway	Official Symbol	Product Protein	Location (Chr)	Length (bp)	Number of SNPs after QC
KP	AADAT	Aminoadipate aminotransferase	4	31,450	7
KP	ACMSD	Aminocarboxymuconate semialdehyde decarboxylase	2	63,610	10
KP	AFMID	Arylformamidase	17	20,384	3
KP	HAAO	3-hydroxyanthranilate 3,4-dioxygenase	2	25,524	7
KP	IDO1	Indoleamine 2,3-dioxygenase 1	8	14,981	4
KP	IDO2	Indoleamine 2,3-dioxygenase 2	8	81,436	35
KP	KMO	Kynurenine 3-monooxygenase	1	63,625	33
KP	KYAT1	Kynurenine aminotransferase 1	9	48,962	4
KP	KYAT3	Kynurenine aminotransferase 3	1	57,187	5
KP	KYNU	Kynureninase	2	293,388	41
KP	QPRT	Quinolinate phosphoribosyltransferase	16	18,990	3
KP	SLC36A4	Solute carrier family 36 member 4	11	50,245	3
KP	SLC3A2	Solute carrier family 3 member 2	11	32,871	3
KP	SLC7A5	Solute carrier family 7 member 5	16	39,471	7
KP	TDO2	Tryptophan 2,3-dioxygenase	4	16,713	1
NM	BST1	Bone marrow stromal cell antigen 1	4	29,722	14
NM	CD38	CD38 molecule	4	74,904	16
NM	NADK	NAD kinase	1	28,799	2
NM	NADK2	NAD kinase 2, mitochondrial	5	49,690	14
NM	NADSYN1	NAD synthetase 1	11	48,613	10
NM	NMNAT1	Nicotinamide nucleotide adenylyltransferase 1	1	42,575	6
NM	NMNAT2	Nicotinamide nucleotide adenylyltransferase 2	1	170,143	30
NM	NMNAT3	Nicotinamide nucleotide adenylyltransferase 3	3	117,870	18
NM	NMRK1	Nicotinamide riboside kinase 1	9	27,578	8
NM	NMRK2	Nicotinamide riboside kinase 2	19	9347	0
NM	NT5E	5’-nucleotidase ecto	6	45,701	5
NM	QPRT	Quinolinate phosphoribosyltransferase	16	18,990	3

Abbreviations: bp: base pair; chr: chromosome; KP: kynurenine pathway; NM: nicotinate metabolism; QC: quality control; SNP: single nucleotide polymorphism.

**Table 2:** SNPs information and the results for SIRTs & ALDHs.

Official Symbol	Product Protein	Location (Chr)	Length (bp)	Number of SNPs after QC
SIRT1	Sirtuin 1	10	33,733	6
SIRT2	Sirtuin 2	19	21,063	3
SIRT3	Sirtuin 3	11	21,901	3
SIRT4	Sirtuin 4	12	21,469	1
SIRT5	Sirtuin 5	6	40,884	8
SIRT6	Sirtuin 6	19	8454	0
SIRT7	Sirtuin 7	17	6237	1
ALDH1A1	Aldehyde dehydrogenase 1 family member A1	9	52,382	15
ALDH1A2	Aldehyde dehydrogenase 1 family member A2	15	112,282	18
ALDH1A3	Aldehyde dehydrogenase 1 family member A3	15	3,6795	15
ALDH1B1	Aldehyde dehydrogenase 1 family member B1	9	5959	5
ALDH1L1	Aldehyde dehydrogenase 1 family member L1	3	77,616	25
ALDH1L2	Aldehyde dehydrogenase 1 family member L2	12	64,668	19
ALDH2	Aldehyde dehydrogenase 2	12	50,599	2
ALDH3A1	Aldehyde dehydrogenase 3 family member A1	17	10,314	9
ALDH3A2	Aldehyde dehydrogenase 3 family member A2	17	29,460	3
ALDH3B1	Aldehyde dehydrogenase 3 family member B1	11	20,726	6
ALDH3B2	Aldehyde dehydrogenase 3 family member B2	11	19,069	9
ALDH4A1	Aldehyde dehydrogenase 4 family member A1	1	31,125	4
ALDH5A1	Aldehyde dehydrogenase 5 family member A1	6	42,238	14
ALDH6A1	Aldehyde dehydrogenase 6 family member A1	14	27,606	6
ALDH7A1	Aldehyde dehydrogenase 7 family member A1	5	53,378	24
ALDH8A1	Aldehyde dehydrogenase 8 family member A1	6	32,708	5
ALDH9A1	Aldehyde dehydrogenase 9 family member A1	1	36451	6
ALDH16A1	Aldehyde dehydrogenase 16 family member A1	19	17,825	0
ALDH18A1	Aldehyde dehydrogenase 18 family member A1	10	50,770	9

Abbreviations: bp: base pair; chr: chromosome; QC: quality control; SNP: single nucleotide polymorphism.

**Table 3:** Information of significant genes and SNPs in tryptophan catabolism and nicotinate metabolism.

Pathway	Gene	SNP	Empirical *p*-Value	*p*-Value	Minor Allele	Major Allele	MAF in Cases	MAF in Controls
Tryptophan catabolism	ACMSD	rs12622574	0.0256	0.0035	A	G	0.1562	0.2078
Nicotinate metabolism	BST1	rs28532698	0.0476	0.0124	C	T	0.0883	0.0607
	CD38	rs3733593	0.0230	0.0017	A	G	0.2437	0.3074

Abbreviations: SNP: single nucleotide polymorphism; MAF: minor allele frequency.

**Table 4:** Haplotype Analysis results of significant SNPs.

Gene	SNP	Block Haplotype	Chi Square	*p*-Value	Permutation *p*-Value
ACMSD	rs12622574	TTTACATT	8.073	0.0045	0.0256
BST1	rs28532698	GCGCC	6.247	0.0124	0.1046
		GC	7.887	0.0050	0.0476
CD38	rs3733593	TCA	9.347	0.0022	0.0230
		TCG	6.122	0.0134	0.1060

Abbreviations: SNP: single nucleotide polymorphism.

## Data Availability

The data are not publicly available due to privacy issues.

## Supplementary Materials

The following supporting information can be downloaded at: https://www.mdpi.com/article/10.3390/jcm11133622/s1. Table S1: SNPs information for kynurenine pathway; Table S2: SNP information for nicotinate metabolism; Table S3: SNPs information and the results for SIRTs; Table S4: SNPs information and the results for ALDHs.
