# Supplementary material for: Identification of Genetic Variations in the NAD-Related Pathways for Patients with Major Depressive Disorder: A Case-Control Study in Taiwan
Source: J Clin Med. 2022 Jun 23;11(13):3622. doi: 10.3390/jcm11133622 (PMC9267440; doi:10.3390/jcm11133622)
Supplement: Supplementary file 1 [file jcm-11-03622-s001.zip › jcm-1712077-supplementary.pdf]

Supplementary Table S1 SNPs information for kynurenine pathway

Gene	SNP	Variation Type	Allele frequency in East Asain[1]
AADAT	rs3796526	intron	0.0770
	rs13145318	Intron	0.6619
	rs963660	intron	0.4459
	rs56358114	intron	0.2815
	rs72702395	intron	0.1888
	rs62344720	intron	0.1892
	rs76766179	Intron	0.7024
ACMSD	rs17322446	intron	0.0739
	rs1893396	intron	0.8940
	rs4954187	intron	0.8927
	rs1942052	intron	0.0739
	rs12622574	intron	0.1870
	rs3819121	intron	0.4646
	rs16831124	intron	0.2911
	rs7564746	intron	0.1362
	rs6711390	intron	0.6015
	rs2166480	intron	0.6219
AFMID	rs8082252	intron	0.6400
	rs8082142	intron	0.6399
	rs8070127	intron	0.2250
HAAO	rs2304661	3' UTR/non coding transcript exon	0.0482
	rs13016201	intron	0.0482
	rs12613026	intron	0.7031
	rs3816183	intron	0.5135
	rs12998043	intron	0.7783
	rs13026205 (A-C)	intron	0.7776
	rs13026205 (A-T)	intron	0.0006
	rs72613891 (C-T)	Intron	0.0000
	rs72613891 (C-G)	intron	0.5350
IDO1	rs7820268	intron	0.2510
	rs7010461 (C-T)	intron/non coding transcript exon	0.3043
	rs7010461 (C-G)	intron/non coding transcript exon	0.0000
	rs62512635	intron	0.2824
	rs3739319	intron	0.5508
IDO2	rs7846217	intron	0.8832

	rs16888382	intron	0.3184
	rs11777027	intron	0.8877
	rs11786337	intron	0.5849
	rs7845003	intron	0.0038
	rs3927941	intron	0.3158
	rs56055654	intron	0.3116
	rs12542808	intron	0.3135
	rs28666749 (A-G)	intron	0.5458
	rs28666749 (ATG-A)	intron	0.0000
	rs2543041	intron	0.8952
	rs28631334	intron	0.0895
	rs6996031	intron	0.3182
	rs79682491	intron	0.3215
	rs60895952	intron	0.2716
	rs2543058	intron	0.4360
	rs1362846 (A-C)	intron	0.2400
	rs1362846 (A-T)	intron	0.0000
	rs1035280	intron	0.6093
	rs10504014	intron	0.2503
	rs7016315	intron	0.2471
	rs16888478	intron	0.7030
	rs61123871	intron	0.6999
	rs7820968	intron	0.3571
	rs2543073	intron	0.5515
	rs1421215	intron	0.3398
	rs888444	intron	0.9114
	rs10109853	missense/intron	0.2332
	rs2909333	intron	0.2358
	rs7017498	intron	0.2356
	rs11435188 (G-GT)	intron	0.3191
	rs11435188 (G-T)	intron	0.0000
	rs10958579	intron	0.3398
	rs2981155	intron	0.7314
	rs2955893	intron	0.6016
	rs57431511	intron	0.2190
	rs4321987	intron	0.3677
	rs2955903	intron	0.3263
KMO	rs3014573 (T-A)	intron	0.0000

	rs3014573 (T-G)	intron	0.0636
	rs10926510	intron	0.1343
	rs3014572	intron	0.1943
	rs75172809	intron	0.0431
	rs1932439 (G-1)	intron	0.0000
	rs1932439 (G-C)	intron	0.4632
	rs1932440	intron	0.8504
	rs1932441	intron	0.7853
	rs4509566	intron	0.1759
	rs2050512	intron	0.6085
	rs2050515	intron	0.2914
	rs1335899	intron	0.1472
	rs4518887	intron	0.1472
	rs12731674	intron	0.1468
	rs6661244	intron	0.7941
	rs77742045	intron	0.2670
	rs10926515	intron	0.1478
	rs2050505	intron	0.2580
	rs11808201	intron	0.2246
	rs61825639	intron	0.0811
	rs3007743 (A-G)	intron	0.0027
	rs3007743 (A-AG)	intron	0.0000
	rs6658805	intron	0.4416
	rs397960509	intron	0.4469
	rs3007736	intron	0.3979
	rs74443223	intron	0.2022
	rs3753214	intron	0.1808
	rs12139931 (T-G)	intron	0.2890
	rs12139931 (T-A)	intron	0.0000
	rs9970943	intron	0.7712
	rs4660103	intron	0.2201
	rs12030098	intron	0.1877
	rs685722	intron	0.8286
	rs10926523	intron	0.1865
	rs655970	intron	0.4186
	rs1053230	missense	0.0533
KYAT1	rs60239222 (C-G)	intron	0.0000
	rs60239222 (C-T)	intron	0.0635

	rs10760581	intron	0.7111
	rs941960	intron	0.7150
	rs2417133	intron	0.0623
KYAT3	rs60436973 (A-T)	intron	0.0000
	rs60436973 (A-C)	intron	0.0985
	rs1325923	intron	0.4178
	rs2765524	intron	0.7103
	rs7526419	intron	0.4172
	rs76764652	intron	0.0583
KYNU	rs7586571	intron	0.2641
	rs1439876	intron	0.5548
	rs1438266	intron	0.6036
	rs17805075	intron	0.2471
	rs6429990	intron	0.7741
	rs16858223	intron	0.2391
	rs11692509	intron	0.3383
	rs3768853	intron	0.0927
	rs13030111	intron	0.1470
	rs9917201	intron	0.3563
	rs351696	intron	0.5862
	rs164735	intron	0.0863
	rs12997823	intron	0.4730
	rs13024113	intron	0.4587
	rs351685 (A-G)	intron	0.0006
	rs351685 (A-C)	intron	0.4487
	rs351684	intron	0.5830
	rs7561260	intron	0.4058
	rs164605	intron	0.5478
	rs10928161	intron	0.2294
	rs1866619	intron	0.8045
	rs10928162	intron	0.2294
	rs62169913	intron	0.2294
	rs6429997	intron	0.7162
	rs76494682	intron	0.1983
	rs4609990	intron	0.1985
	rs62169914	intron	0.2406
	rs10496933	intron	0.2274
	rs1371516	intron	0.4719

	rs11902056	intron	0.5065
	rs34332680	intron	0.2458
	rs352873	intron	0.2532
	rs352889	intron	0.5386
	rs352887	intron	0.7826
	rs189526	intron	0.2526
	rs16858506	intron	0.2432
	rs352922	intron	0.5181
	rs10496939	intron	0.7755
	rs6430001 (A-G)	intron	0.0000
	rs6430001 (A-C)	intron	0.4833
	rs34550924	intron	0.2571
	rs73961708	intron	0.2593
	rs9013	missense	0.2769
QPRT	rs12596308	intron	0.5870
	rs9923341	intron	0.1122
	rs2303255	missense/intron	0.0676
SLC3A2	rs10897300	intron	0.1391
	rs489381 (A-G)	intron	0.8322
	rs489381 (A-C)	intron	0.0000
	rs4726	synonymous/5' UTR/ non coding transcript exon/3' UTR	0.2009
SLC7A5	rs1060253	intron	0.5811
	rs747578	intron	0.5584
	rs11865049	intron	0.1308
	rs1060250	missense/5' UTR	0.0001
	rs8052746	intron	0.8514
	rs67288628	intron	0.6619
	rs876985	intron	0.1677
SLC36A4	rs10830993	intron	0.4200
	rs10831001	intron	0.4303
	rs10831005	intron	0.3130
TDO2	rs2878125	intron	0.9069

Note: [1]Data from gnomAD human genome database; [2]rs397960509 was merged into rs11415825 on October 11, 2018

Supplementary Table S2 SNP information for nicotinate metabolism

Gene	SNP	Variation Type	Allele frequency in East Asain[1]
BST1	rs2302467	intron	0.7136
	rs3756246	intron	0.3534
	rs2302465	missense	0.0343
	rs2302464	missense	0.2088
	rs16892260	intron	0.4664
	rs2302463	intron	0.1876
	rs3213710	intron	0.4355
	rs60938249	intron	0.2416
	rs28532698	intron	0.0686
	rs12643475	intron	0.0680
	rs9942275	intron	0.2706
	rs9291637	intron	0.3744
	rs76228166	intron	0.1600
	rs1058212	synonymous/intron	0.5133
CD38	rs3756242	intron	0.1431
	rs6449190	intron	0.4349
	rs7679308	intron	0.2416
	rs13104011	intron	0.1427
	rs16892418	intron	0.1923
	rs6836946	intron	0.3740
	rs6841880	intron	0.3721
	rs7655635	intron	0.1440
	rs6449195	intron	0.2288
	rs4698121	intron	0.1430
	rs16892438	intron	0.1928
	rs10805347	intron	0.4317
	rs1130169	3’ UTR	0.2765
	rs17476066	3’ UTR	0.2033
	rs4396987	3’ UTR	0.1913
	rs3733593	3’ UTR	0.3082
NADK	rs34306661	intron/non coding transcript exon	0.2551
	rs7531583	intron	0.6789
NADK2	rs7716788	intron	0.2789
	rs16902801	intron	0.1060
	rs7723157	intron	0.0803

	rs13174907 (C-T)	intron	0.0799
	rs13174907 (C-A)	intron	0.0000
	rs41445147	intron	0.6987
	rs78923858	intron	0.1871
	rs991032	intron	0.6540
	rs79218367	intron	0.2195
	rs75663322	intron	0.2175
	rs76901139	intron	0.2181
	rs10079850	intron	0.4806
	rs10941279	intron	0.2806
	rs79914640	intron	0.1190
	rs10075150	intron	0.3902
NADSYN1	rs12785878 (G-T)	intron	0.4103
	rs12785878 (G-A)	intron	0.0006
	rs7938885	intron/non coding transcript exon	0.4240
	rs12800438	intron	0.4093
	rs1792225	intron	0.8389
	rs3794060	intron/3' UTR	0.4087
	rs3829251	intron	0.2893
	rs7952035	intron	0.0784
	rs949178	intron	0.8387
	rs4944998	intron	0.7010
	rs10898210	intron/non coding transcript exon	0.7616
NMNAT1	rs12064822	intron	0.2219
	rs1220421	intron	0.6141
	rs12062953	intron/non coding transcript exon	0.2193
	rs12071307	intron	0.2219
	rs2890358	intron	0.6150
	rs6683452	intron	0.8387
NMNAT2	rs41304261	3' UTR	0.0657
	rs10911291	intron	0.1928
	rs77536160 (C-T)	intron	0.0675
	rs77536160 (C-A)	intron	0.0000
	rs10911295	intron	0.1928
	rs10494561	intron	0.1947
	rs500530	intron	0.2778
	rs588492	intron	0.5141
	rs17541993	intron	0.0500

	rs10752907	intron	0.3694
	rs7545584	intron	0.5245
	rs16860763	intron	0.3811
	rs6687056	intron	0.0904
	rs117940031	intron	0.0776
	rs2276879	intron	0.3793
	rs602182	intron	0.3247
	rs16860791	intron	0.0000
	rs10911305	intron	0.8164
	rs12045638	intron	0.0814
	rs543423	intron	0.8956
	rs12130199	intron	0.7934
	rs1330224	intron	0.7941
	rs581606	intron	0.1205
	rs944190	intron	0.8755
	rs481157	intron	0.9169
	rs664422	intron	0.8036
	rs10911319	intron	0.1864
	rs542349	intron	0.9160
	rs673593	intron	0.6828
	rs2485932	intron	0.6765
	rs1361197	intron	0.6772
NMNAT3	rs76015646	intron	0.0899
	rs12485554	intron	0.2202
	rs10935338	intron	0.3887
	rs295464	intron	0.3524
	rs47442	intron	0.3528
	rs9835596	intron	0.2192
	rs1021600	intron	0.8447
	rs10804666	intron	0.7371
	rs12635533	intron	0.6001
	rs17314168	intron	0.3428
	rs16849222	intron	0.3355
	rs2292412	intron	0.6967
	rs2291802	intron	0.2384
	rs4683613	intron	0.8312
	rs11718568	intron	0.2362
	rs11714387	intron	0.4356

	rs2349591	intron	0.2990
	rs9815708	intron	0.2219
NMRK1	rs7852863	intron	0.8032
	rs7021926	intron	0.7933
	rs13289688	intron/3’ UTR	0.7752
	rs3752955	synonymous/intron/3’ UTR	0.6915
	rs12686433	intron	0.1799
	rs17060790	intron	0.1538
	rs11144217	intron	0.7861
	rs11144218	intron	0.5605
NT5E	rs17819627 (C-A)	intron	0.0000
	rs17819627 (C-T)	intron	0.1049
	rs6942065	intron	0.0641
	rs4235826	intron	0.5725
	rs753264245 (G-A)	intron	0.0000
	rs753264245 (G-C)	intron	0.0006
	rs9450284 (G-T)	intron	0.0000
	rs9450284 (G-C)	intron	0.5274
QPRT	rs12596308	intron	0.5870
	rs9923341	intron	0.1122
	rs2303255	missense/intron	0.0676

Note: [1]Data from gnomAD human genome database

Supplementary Table S3 SNPs information and the results for SIRTs

Gene	SNP	Variation Type	Allele frequency in East Asain[1]
SIRT1	rs7096385 (T-C)	intron	0.7017
	rs7096385 (T-G)	intron	0.0000
	rs2273773	synonymous/non coding transcript exon	0.2897
	rs10823112	intron	0.2999
	rs16924945	intron	0.4272
	rs74790878	intron	0.4303
	rs4746720	3’ UTR	0.4292
SIRT2	rs7251489	intron	0.3581
	rs3136578	intron	0.8558
	rs11575003	intron	0.1067
SIRT3	rs72770975	intron	0.0940
	rs11250663	intron	0.1504
	rs2436024	intron	0.9403
SIRT4	rs16950058	synonymous/non coding transcript exon	0.1041
SIRT5	rs2841506	intron	0.0986
	rs9382227	intron	0.2160
	rs2804910	intron	0.1004
	rs2804911 (A-T)	5’ UTR	0.0000
	rs2804911 (A-C)	5’ UTR	0.1008
	rs2804912 (T-A)	intron	0.0000
	rs2804912 (T-G)	intron	0.3019
	rs4712047	intron	0.5551
	rs2804921	intron	0.0506
	rs3757261	synonymous/3’ UTR	0.1726
SIRT7	rs552020219	missense/non coding transcript exon	0.0008

Note: [1]Data from gnomAD human genome database

Supplementary Table S4 SNPs information and the results for ALDHs

Gene	SNP	Variation Type	Allele frequency in East Asain[1]
ALDH1A1	rs4646547	intron	0.5668
	rs79539727	intron	0.0591
	rs1888202	intron	0.4383
	rs63319	intron	0.5155
	rs348457	intron	0.1568
	rs4646544 (G-T)	intron	0.0650
	rs4646544 (G-A)	intron	0.0006
	rs8187929	missense/non coding transcript exon	0.0418
	rs2288086	intron	0.0667
	rs1049981	missense	0.0000
	rs4237255	intron	0.4632
	rs13959	synonymous/non coding transcript exon	0.4662
	rs76100846	intron	0.0564
	rs1424482	intron	0.4277
	rs8187876	intron	0.4749
	rs4646540	intron	0.0469
ALDH1A2	rs3784264	intron	0.6866
	rs3784260	intron	0.1986
	rs4646612 (C-G)	intron	0.0000
	rs4646612 (C-T)	intron	0.1949
	rs12898976	intron	0.3215
	rs16977883	intron	0.1240
	rs1899355	intron	0.6778
	rs78794515	intron	0.1227
	rs79462598	intron	0.1243
	rs61999891	intron	0.2136
	rs4646592	intron	0.3239
	rs4646583	intron	0.1232
	rs2642632	intron	0.3194
	rs74522619	intron	0.1242
	rs4144005	intron	0.3255
	rs11071365	intron	0.1956
	rs4238327	intron	0.3211
	rs4646572	intron	0.1961
	rs12914603	intron	0.3196

ALDH1A3	rs3803433	3’ UTR/intron	0.6218
	rs4646649	intron	0.5724
	rs12905372	intron	0.5032
	rs4646675	intron	0.7346
	rs4646678	intron	0.4567
	rs4646680	intron	0.0824
	rs3825926	intron	0.0700
	rs3825925	intron	0.0783
	rs3803430	missense/non coding transcript exon	0.0632
	rs3825924	splice region	0.5408
	rs4246328	intron	0.6600
	rs4646683	intron	0.0699
	rs11854028 (G-T)	intron	0.7182
	rs11854028 (G-A)	intron	0.0000
	rs6598396	intron	0.7307
	rs14226	3’ UTR	0.4550
ALDH1B1	rs4646769	intron	0.7864
	rs4646770	intron	0.2777
	rs2228093 (C-T)	missense	0.3799
	rs2228093 (C-G)	missense	0.0000
	rs2073478 (G-T)	missense	0.3146
	rs2073478 (G-A)	missense	0.0000
	rs3043 (G-C)	3’ UTR	0.7031
	rs3043 (G-A)	3’ UTR	0.0000
ALDH1L1	rs1127717	missense/3’ UTR/intron	0.1329
	rs62263532	intron	0.1508
	rs11924478	intron	0.2513
	rs3772421	intron	0.5238
	rs3821464	intron	0.5736
	rs4646726	intron	0.3736
	rs3772413	intron	0.2041
	rs2305230	synonymous/3’ UTR/ non coding transcript exon	0.2128
	rs7639966	intron	0.5760
	rs13089821	intron	0.3752
	rs2886059	missense/ non coding transcript exon/ 3’ UTR	0.2115
	rs10934749	intron	0.5636

	rs4646703	intron	0.1248
	rs1868138	intron	0.3194
	rs1823213	intron	0.7105
	rs4646701	intron	0.4961
	rs13093423	intron	0.6546
	rs4521172	intron	0.4370
	rs4679102	intron	0.6604
	rs16837189	intron	0.2326
	rs4646700	intron	0.4416
	rs4646699	intron	0.2156
	rs7617733	intron	0.2461
	rs9842910	intron	0.2413
	rs13060596	intron	0.4096
ALDH1L2	rs7313529	3' UTR/non coding transcript exon	0.2702
	rs76906408	intron	0.0763
	rs11112317	intron	0.0566
	rs12372636	intron	0.0565
	rs4964318	intron	0.7602
	rs2228700	intron	0.4146
	rs7301423	intron	0.4884
	rs11112341	intron	0.4212
	rs11112346	intron/non coding transcript exon	0.2024
	rs73393835 (A-T)	intron	0.0000
	rs73393835 (A-G)	intron	0.0603
	rs12581638	intron	0.3997
	rs10861340	intron	0.2981
	rs7299166	intron	0.9365
	rs7134351	intron	0.3044
	rs10459204	intron	0.2413
	rs10459164	intron	0.2442
	rs1345094	intron	0.6421
	rs77352169	intron	0.0628
	rs73393881	intron	0.0551
ALDH2	rs13306164	synonymous/intron/3' UTR	0.0248
	rs671	missense/3' UTR	0.2554
ALDH3A1	rs57555435	synonymous/missense	0.1138
	rs2228100	missense/intron/<br>non coding transcript exon	0.4330

	rs3744692	missense/intron/<br>non coding transcript exon	0.0573
	rs60739355	missense/intron/<br>non coding transcript exon	0.0000
	rs2072330	synonymous/non coding transcript exon	0.3072
	rs57633837	synonymous/non coding transcript exon	0.0000
	rs887241 (A-C)	missense/non coding transcript exon	0.9480
	rs887241 (A-T)	missense/non coding transcript exon	0.0000
	rs4646787	intron	0.9544
	rs2072327	5’ UTR/intron	0.4523
ALDH3A2	rs16960280	intron	0.2291
	rs12937946	intron	0.5501
	rs7215	3’ UTR/non coding transcript exon	0.9243
ALDH3B2	rs3741172	missense/synonymous	0.0883
	rs1551886	missense/synonymous	0.9117
	rs17856219	missense/non coding transcript exon	0.0884
	rs4930465	intron	0.9128
	rs4646823	5’ UTR/non coding transcript exon	0.8839
	rs34589365	intron	0.8887
	rs1871031	intron	0.7706
	rs61894527	intron	0.0873
	rs17510687	intron	0.0874
ALDH4A1	rs2230708	synonymous	0.7107
	rs6426802	intron	0.7128
	rs2230705 (C-G)	synonymous	0.4492
	rs2230705 (C-T)	synonymous	0.0000
	rs16862344	intron	0.0647
ALDH5A1	rs2010190	intron	0.6250
	rs2245674	intron	0.1928
	rs2760118	missense	0.1863
	rs3765310	missense	0.0807
	rs3765311	intron	0.4193
	rs2760117	intron	0.1898
	rs2817220	splice region	0.1005
	rs2328824	intron	0.4308
	rs1569579	intron	0.5441
	rs807515	intron	0.5486
	rs11751325	intron	0.3022

	rs809419	intron	0.3458
	rs2744597	intron	0.4851
	rs2744598	intron	0.1911
ALDH6A1	rs2072293	intron/non coding transcript exon	0.8335
	rs4243645	intron/non coding transcript exon	0.9095
	rs12884055	intron	0.0701
	rs4646861	intron/non coding transcript exon	0.5931
	rs62005121	intron	0.2122
	rs4646858	intron	0.9094
ALDH7A1	rs1060856	3’ UTR	0.1465
	rs2775	3’ UTR/non coding transcript exon	0.3477
	rs4240406	intron	0.4664
	rs11241901	intron	0.4489
	rs4836272	intron	0.6010
	rs13179664	intron	0.5870
	rs1038378	intron	0.5032
	rs1038381	intron	0.0805
	rs4379190	intron	0.7529
	rs12514417	missense/splice region/ non coding transcript exon	0.1430
	rs2306618	missense/non coding transcript exon	0.0809
	rs78434690	intron	0.1037
	rs4835913	intron	0.5974
	rs4836276	intron/non coding transcript exon	0.5354
	rs4626334	intron/non coding transcript exon	0.7748
	rs4626335	intron/non coding transcript exon	0.4320
	rs28429591	intron	0.1716
	rs2035472	intron	0.8665
	rs4836277	intron	0.8549
	rs7709115	intron	0.1499
	rs13182402	intron	0.0527
	rs75640104	intron	0.0759
	rs6595719	intron	0.1647
	rs34241005	intron	0.2174
ALDH8A1	rs2294321	intron	0.7700
	rs3817776	intron	0.5135
	rs7772031	intron	0.7699
	rs7754722	intron	0.7716

	rs3813342 (C-T)	synonymous	0.0000
	rs3813342 (C-A)	synonymous	0.2717
ALDH9A1	rs7534437	intron	0.1667
	rs6683969	intron	0.1710
	rs10800130	intron	0.9454
	rs7546215	intron	0.3678
	rs10800133	intron	0.3669
	rs4646883	5' UTR/intron/non coding transcript exon	0.5696
ALDH18A1	rs7534437	intron	0.1667
	rs6683969	intron	0.1710
	rs10800130	intron	0.9454
	rs7546215	intron	0.3678
	rs10800133	intron	0.3669
	rs4646883	5' UTR/intron/non coding transcript exon	0.5696
	rs7534437	intron	0.1667
	rs6683969	intron	0.1710
	rs10800130	intron	0.9454

Note: [1]Data from gnomAD human genome database
